# Age-dependent molecular variations in osteosarcoma: implications for precision oncology across pediatric, adolescent, and adult patients

**DOI:** 10.3389/fonc.2024.1382276

**Published:** 2024-05-22

**Authors:** Changye Zou, Renxuan Huang, Tiao Lin, Yaxian Wang, Jian Tu, Liwen Zhang, Bo Wang, Jintao Huang, Zhiqiang Zhao, Xianbiao Xie, Gang Huang, Kai Wang, Junqiang Yin, Jingnan Shen

**Affiliations:** ^1^ Department of Musculoskeletal Oncology Center, The First Affiliated Hospital of Sun Yat-sen University, Guangzhou, China; ^2^ OrigiMed, Shanghai, China

**Keywords:** osteosarcoma, pediatrics, adolescents, adults, genomic alteration, next generation sequencing

## Abstract

**Background:**

Osteosarcoma is a leading subtype of bone tumor affecting adolescents and adults. Comparative molecular characterization among different age groups, especially in pediatric, adolescents and adults, is scarce.

**Methods:**

We collected samples from 194 osteosarcoma patients, encompassing pediatric, adolescent, and adult cohorts. Genomic analyses were conducted to reveal prevalent mutations and compare molecular features in pediatric, adolescent, and adult patients.

**Results:**

Samples from 194 osteosarcoma patients across pediatric to adult ages were analyzed, revealing key mutations such as TP53, FLCN, NCOR1, and others. Children and adolescents showed more gene amplifications and HRD mutations, while adults had a greater Tumor Mutational Burden (TMB). Mutations in those over 15 were mainly in cell cycle and PI3K/mTOR pathways, while under 15s had more in cell cycle and angiogenesis with higher VEGFA, CCND3, TFEB mutations. CNV patterns varied with age: VEGFA and XPO5 amplifications more in under 25s, and CDKN2A/B deletions in over 25s. Genetic alterations in genes like MCL1 and MYC were associated with poor prognosis, with VEGFA mutations also indicating worse outcomes. 58% of patients had actionable mutations, suggesting opportunities for targeted therapies. Age-specific patterns were observed, with Multi-TKI mutations more common in younger patients and CDK4/6 inhibitor mutations in adults, highlighting the need for personalized treatment approaches in osteosarcoma. In a small group of patients with VEGFR amplification, postoperative treatment with multi-kinase inhibitors resulted in a PR in 3 of 13 cases, especially in patients under 15. A significant case involved a 13-year-old with a notable tumor size reduction achieving PR, even with other genetic alterations present in some patients with PD.

**Conclusion:**

This study delineates the molecular differences among pediatric, adolescent, and adult osteosarcoma patients at the genomic level, emphasizing the necessity for precision diagnostics and treatment strategies, and may offer novel prognostic biomarkers for patients with osteosarcoma. These findings provide a significant scientific foundation for the development of individualized treatment approaches tailored to patients of different age groups.

## 1 Introduction

Osteosarcoma, a relatively uncommon malignancy, manifests a distinct incidence pattern characterized by a significant surge during the growth phase of adolescence. The rarity of this disease poses substantial challenges for conducting broad research initiatives, rendering large-scale studies especially crucial (1, 2). Despite significant advances in limb-salvage surgery and multimodal chemotherapy, the long-term survival for non-metastatic osteosarcoma has stagnated around 70% for the past few decades, while patients presenting with metastatic disease at diagnosis have an even poorer prognosis, with survival rates hovering around 20-30% (3, 4). Moreover, the high-dose chemotherapeutic regimens necessary for treatment are associated with considerable acute and long-term morbidity (5, 6).

The complexity of osteosarcoma is underscored by its clinical and molecular heterogeneity, which poses a challenge for the identification of prognostic markers and therapeutic targets. Clinical prognosticators, including tumor size, site, and response to chemotherapy, have been well-documented (7). In multivariate analysis, proximal position within the limb was independently associated with worse overall survival of patients with osteosarcoma (8). Previous study showed that the prognosis of male osteosarcoma is slightly worse than that of female patients (9). Age is generally considered to be an independent factor of osteosarcoma. Due to the onset characteristics of osteosarcoma, that is, patients with osteosarcoma show a bimodal distribution according to age, with the highest proportion of 15-19 years old, followed by 75-79 years old, and then 25-59 years old (10). The study found that in adolescent patients with osteosarcoma, the younger the age, the worse the prognosis (7). However, the impact of age on disease characteristics and outcomes has become a focal point of recent research, with studies suggesting that older age may correlate with a higher frequency of adverse histologic response to chemotherapy and inferior survival (11, 12).

Advancements in high-throughput sequencing technologies have revolutionized our understanding of osteosarcoma biology, revealing a multitude of genetic alterations with potential prognostic and therapeutic implications (13). Unlike other solid tumors, osteosarcoma has a low proportion of common driver gene mutations and depends more on the activation of signal pathways caused by gene amplification or overexpression, such as PI3K/mTOR (14), IGF1 (15), VEGF (16), and PDGF (17), etc. Clinical trials of molecular typing and targeted therapy of osteosarcoma based on gene mutations are particularly necessary to improve the prognosis of patients with osteosarcoma. Due to the low incidence rate of osteosarcoma and the heterogeneity of osteosarcoma, it is difficult to carry out large-scale clinical trials. The clinical characteristics of osteosarcoma in young and elderly patients have been reported to differ, along with correlations between clinical features and prognosis (7, 18–20). A few studies have also depicted the distinct genomic profiles of osteosarcomas in different age groups, revealing age-specific mutation patterns and alterations in signaling pathways (14, 15). These findings underline the significance of age as a biological variable in osteosarcoma and may pave the way for age-specific treatment strategies. In a glioma study, age groups defined by 15 and 25 years - children (<15 years), adolescents (15-25 years), and adults (>25 years) - were found to have distinct molecular features, such as differences in the proportion of IDH1,BRAF and H3G34 mutations (21).

Studies have shown that in untreated osteosarcoma, the presence of Vascular Endothelial Growth Factor (VEGF) serves as a prognostic indicator for the likelihood of pulmonary metastasis and unfavorable outcomes in patients undergoing intensive therapy (22). Additionally, a notable discovery is that patients who are very young exhibit a substantially increased incidence of capillary hemangioma-like histological features (10.2% compared to 2.9%; P = 0.017) (23). These insights provide a crucial foundation for devising therapeutic approaches that concentrate on the vascular growth properties of osteosarcoma and may also prompt a reconsideration of age ranges for the inclusion of subjects in clinical trials for emerging therapeutics.

This study is designed to conduct a comprehensive analysis of the molecular mutation characteristics in osteosarcoma patients across various age groups, with the intent of identifying unique molecular markers that could enable personalized treatment approaches. By delving into the age-specific mutation patterns and signaling pathway alterations as previously reported, and utilizing advanced genomic data, our goal is to enhance the current understanding of the complex molecular landscape of osteosarcoma. Ultimately, this research aims to contribute to precision medicine efforts, and thereby improve clinical outcomes for osteosarcoma patients of all ages.

## 2 Patients and methods

### 2.1 Patients and samples

From June 2017 to April 2023, we have gathered a cohort of 194 osteosarcoma patients from The First Affiliated Hospital of Sun Yat-sen University. These patients were selected based on their visits to our institution for treatment, during which they also underwent genetic testing. These patients were stratified into three age groups for analysis: pediatric patients (under 15 years), adolescent and young adult patients (15 - 25 years), and adult patients (over 25 years). Samples included fresh surgical/biopsy tissues or formalin-fixed, paraffin-embedded (FFPE) tumor tissues, along with matched blood samples, which were collected to detect genomic alterations (GAs). Genomic DNA was prepared by using QIAamp DNA FFPE Tissue Kit and QIAamp DNA Blood Midi Kit (Qiagen, Hilden, Germany) according to the manufacturer’s instructions. The concentration of DNA was measured and normalized to 20–50 ng/μL. The study was approved by the Ethics Committee of The First Affiliated Hospital of Sun Yat-sen University, and all patients signed an informed consent form.

### 2.2 Genomic alterations identification

The DNA samples were detected by using the NGS-based Yuansu 450 gene panel or whole exon sequencing (WES) (OrigiMed, Shanghai, China). Yuansu 450gene panel covers all the coding exons of 450 tumor-related genes, the genes were captured and sequenced with a mean depth of 1000×. WES libraries were prepared and captured using the SureSelect Human All Exon V6 kit (Agilent Technologies) following manufacturer’s instructions and sequenced with a mean coverage depth of 300X. All these experiments were performed on Illumina Novaseq 6000 system (Illumina, Inc., CA). Resultant sequences were further analyzed for genomic alterations compared with normal genomic DNA, including single nucleotide variants, short and long insertions/deletions (indels), copy number variations, and structural variants of gene rearrangement/fusion. The tumor mutational burden (TMB) was estimated by analyzing somatic mutations, including coding base substitution and INDELs, per mega-base of the panel sequences examined.

### 2.3 Mutational signature analysis

According to the number of different types of point mutations such as C > A/G > T, C > G/G > C, C > T/G > A, T > A/A > T, T > C/A > G, and T > G/A > C, a cluster analysis was performed in order to observe similarity in tumor samples. Extracted mutational features were compared with the pan-cancer catalogue for 94 known features cited in the cancer somatic mutation catalogue (COSMIC) database (https://cancer.sanger.ac.uk/signatures/) using Mutational Patterns packages (3.6.0) (24). The similarity of mutational features was assessed based on a cosine similarity > 0.85, which indicated common features.

### 2.4 Function enrichment analyses

To explore the biological functions of somatic mutations, gene ontology (GO) and a Kyoto Encyclopedia of Genes and Genomes (KEGG) enrichment analysis were conducted using the ClusterProfiler (v.3.10.1) in the R software.

### 2.5 Statistical analysis

Statistical analysis was conducted using the R statistical software package (R Foundation for Statistical Computing, Vienna, Austria). Categorical variables are expressed as in frequency and percentages; Continuous variables was presented with medians and percentiles. Wilcoxon rank test was used for comparing two continuous data and Fisher’s exact tests were used for comparing two categorical data. P < 0.05 was considered statistically significant.

## 3 Results

### 3.1 Characterization of osteosarcoma patients

A total of 194 osteosarcoma patients were enrolled in this study. There were 127 males and 67 females. The median age was 16 Years old, ranged from 5-73 years old. Osteosarcoma is predominantly seen in adolescents, with about 75% of cases occurring between the ages of 15 and 25. Therefore, we categorize patients aged younger 15 as pediatric patients, those between 15 to 25 years as adolescent & young adult patients, and those over 25 years as adult patients. Our cohort includes 81 pediatric patients, 85 adolescent patients, and 28 adult patients. Based on pathological staging records, there were 2 cases of Stage I tumors, 136 cases of Stage II tumors, 2 cases of Stage III tumors, and 43 cases of Stage IV tumors. Additionally, there were 11 cases of pelvic and spinal bone tumors for which the AJCC staging criteria were not applicable. Within this cohort, the YuanSu450 gene panel and WES (Whole Exome Sequencing) (OrigiMed, Shanghai, China) were utilized to test samples from 110 and 84 patients, respectively. In this cohort, 177 underwent immunohistochemistry testing for PD-L1 expression, there were 44 PD-L1 positive patients and 133 negative patients. The majority of patients (193/194, 99.5%) were characterized as having Microsatellite Stable (MSS) tumors (**Table 1**).

**Table 1:** Patient characteristics.

Characteristics	Total (n = 194)
**Age,n(%), median(min-max)**	16(5-73)
**Age<15**	81 (42)
**15≤Age≤25**	85 (44)
**Age>25**	28 (14)
**Gender, n (%)**	Total (n = 194)
**Female**	66 (34)
**Male**	128 (66)
**Stage, n (%)**	Total (n = 194)
**IA**	1 (0.5)
**IB**	1 (0.5)
**IIA**	44 (23)
**IIB**	92 (47)
**III**	2 (1)
**IVA**	43 (22)
**/**	11 (6)
**Histologic subtypes**	Total (n = 194)
**Parosteal osteosarcoma**	3 (1.5)
**Small cell osteosarcoma**	1 (0.5)
**Conventional osteosarcoma**	190 (98)
**grading**	Total (n = 194)
**G1**	2 (1)
**G2**	192 (99)
**therapy**	Total (n = 194)
**Neoadjuvant + surgery + adjuvant**	164 (85)
**surgery + adjuvant**	30 (15)
**MSI, n (%)**	Total (n = 194)
**MSS**	193 (99)
**MSI-H**	1 (1)
**PD-L1 (≥1=positive)**	Total (n = 177)
**Positive**	44 (25)
**Negative**	133 (75)

In staging patients, the AJCC (American Joint Committee on Cancer) system is utilized. However, it should be noted that for pelvic and spinal bone tumors (n=11), the AJCC staging criteria are not applicable, as indicated by the “/” symbol, according to the Bone Cancer NCCN Guidelines 2024, Version 2.

### 3.2 Mutational profile of osteosarcomas

In this research, a total of 23,951 gene mutations were detected among 194 osteosarcoma patients. Gene panel testing yielded 1,066 mutations in 110 patients, while whole-exome sequencing (WES) detected 22,885 mutations in 84 patients. We found that the most prevalent mutation type in osteosarcomas was gene amplification (71.8%), followed by substitutions/deletions (19.7%), gene fusions (3.8%), homozygous deletions (2.6%), and truncating mutations (2.1%) (**Supplementary Figure S1**).

**Figure 1:**
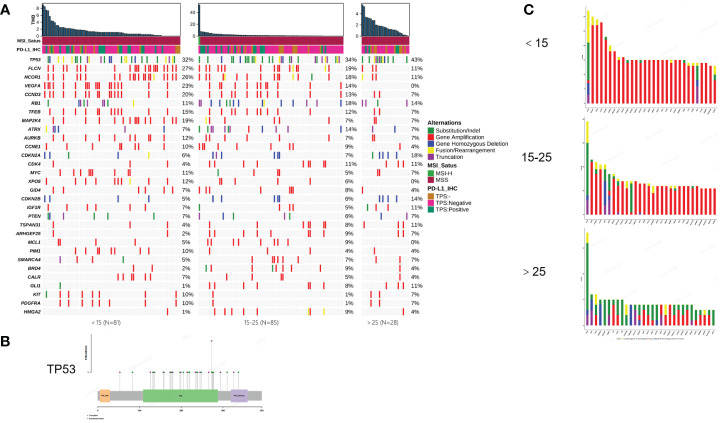
Mutational characterization of this cohort. **(A)** Mutational landscape of 194 osteosarcoma patients (<15, 15-25, >25, respectively). **(B)** Distribution of TP53 mutation sites. **(C)** Amplification statistics of patients in different age groups (< 15, 15-25, > 25, respectively). Green represents substitution/Indel mutations, red represents gene amplification mutations, blue represents gene homozygous deletion mutations, yellow represents fusion/rearrangement mutations, and purple represents truncation mutations.

To illustrate the genomic landscape based on a large cohort, we integrated the results of WES and panel testing by filtering the WES data for panel probe sites. The results revealed that the most frequent mutations in osteosarcomas were in *TP53* (39%), *FLCN* (22%), *NCOR1* (21%), *VEGFA* (16%), *CCND3* (15%), *RB1* (14%), *TFEB* (12%), and *MAP2K4* (11.8%) (**Figure 1A**). *TP53* mutations were predominantly of the substitution/indel type and concentrated in the p53 protein's DNA-binding domain (**Figure 1B**).

### 3.3 Comparison of mutation characteristics between pediatric, adolescent and adult osteosarcoma patients

**Figure 2:**
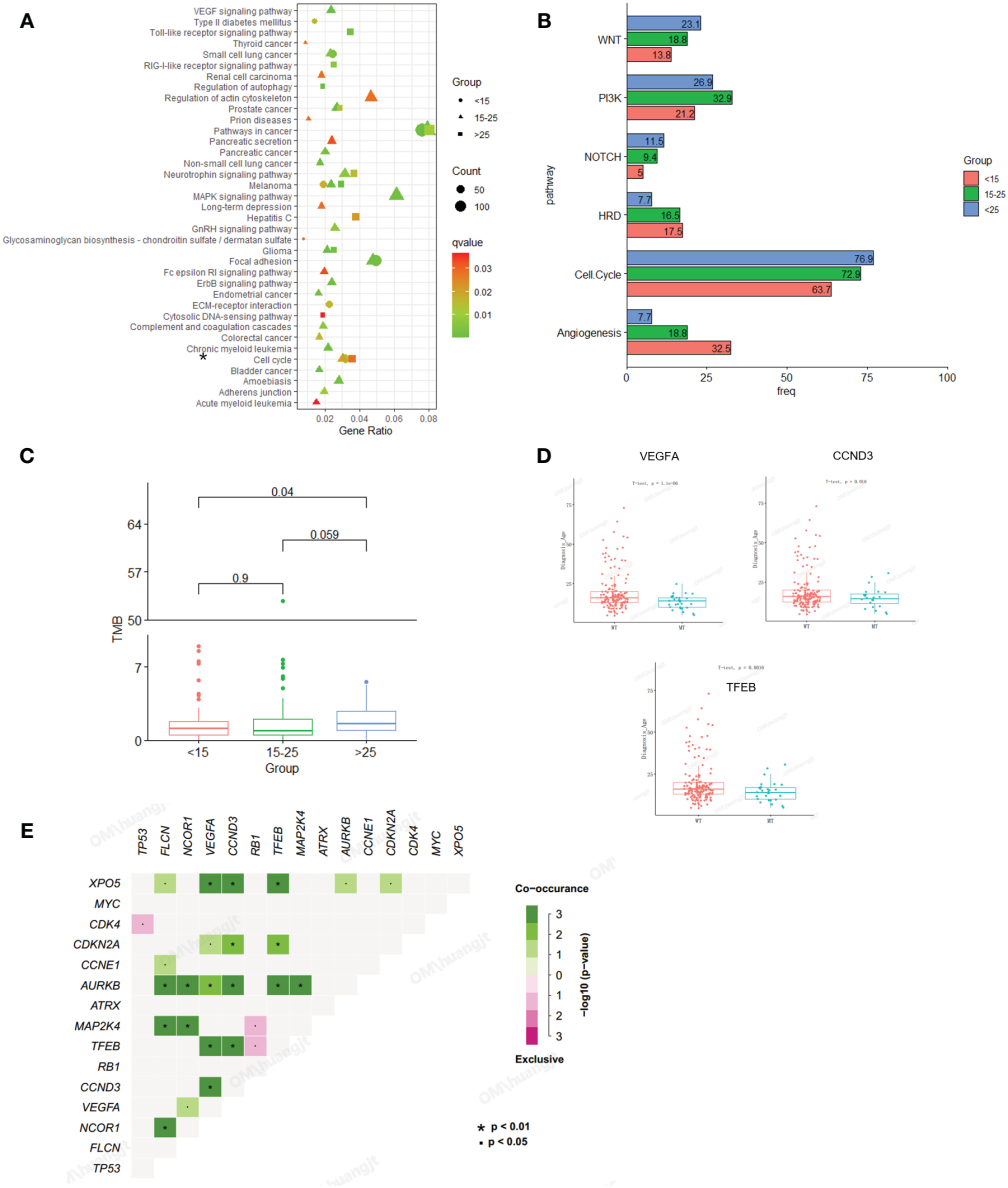
Comparison of mutation characteristics between osteosarcoma subgroups (pediatric, adolescent and adult). **(A)** Enrichment analysis of mutated pathways in adult osteosarcoma group and adolescent osteosarcoma group. From blue to red represents the gradually decreased p value, circle size represents the count of mutations in each pathway. **(B)** Enrichment differences in signaling pathways across age groups. **(C)** Association analysis between TMB and osteosarcoma subgroup (pediatric, adolescent and adult). **(D)** Correlation analysis between genetic variation and age. **(E)** Co-mutation analysis of genes.

In this study, osteosarcoma patients under the age of 15 were categorized as pediatric patients, those aged 15-25 as adolescent patients, and those over the age of 25 as adult patients. Our findings indicate that pediatric and then adolescent patients have a higher frequency of gene amplification compared to adult patients, while substitution/Indel mutation types are less frequent than in adult osteosarcoma patients (**Figure 1C**). Enrichment analysis indicates that gene mutations in patients older than 15 years are mainly enriched in the cell cycle and PI3K/mTOR signaling pathways, while gene mutations in patients younger than 15 years are primarily enriched in the cell cycle and angiogenesis signaling pathways. Additionally, we compared Homologous Recombination Deficiency (HRD) mutated genes. The results showed that the frequency of HRD mutations in pediatric and adolescent patients was higher than in adult patients (17.5% vs. 16.5% vs. 7.7%) (**Figures 2A, B**). We also compared the Tumor Mutational Burden (TMB) distribution among pediatric, adolescent, and adult patients. It was found that the TMB in adult patients was higher than in pediatric patients, with a statistically significant difference (p = 0.04) (**Figure 2C**). Compared with adult patients, the mutation frequency of *VEGFA* (P <0.001), CCND3 (P = 0.016) and *TEEB* (P = 0.0016) were significantly higher in pediatrics and adolescents (**Figure 2D**). The co-mutation analysis revealed that *VEGFA*, *CCND3*, and *TEEB* are the most commonly co-mutated genes. In addition, *XPO5* is frequently co-mutated with *VEGFA*, *CCND3*, and *TEEB*, while *AURKB* is commonly co-mutated with *FLCN*, *NCOR1*, *CCND1*, *TFEB*, and *MAP2K4* (**Figure 2E**).

### 3.4 Analysis of CNV distribution of osteosarcoma in different age groups

**Figure 3:**
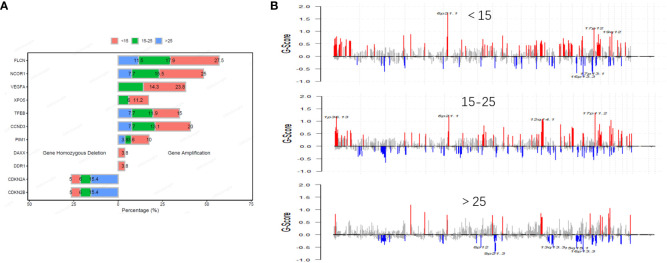
Distribution and frequency of CNVs in osteosarcoma across different age groups. The frequency of common CNV in osteosarcoma is different in different age groups **(A)**. Distribution of CNVs in osteosarcoma across different age groups **(B)**.

In our study, we identified a total of 1,156 copy number variation (CNV) variants across a cohort of 194 patients with osteosarcoma. Among all CNVs, amplifications of *VEGFA* and *XPO5* were primarily found in patients under the age of 25, while amplifications of *DAXX* and *DDR1* were concentrated in patients under the age of 15. Conversely, deletions of *CDKN2A* and *CDKN2B* were more prevalent in the age group over 25 years old (**Figure 3A**). Additionally, within a subset of 75 osteosarcoma patients who underwent whole-exome sequencing (WES), we detected 668 CNV variants. The most frequent CNV include 17p11.2, 19q12, 6q21.1 and 12q12.1 amplification. In 31 pediatric patients, 258 CNVs we detected including 247 amplifications and 11 gene homozygous deletions, and the most frequent CNVs included 6p21.1 amplification, 17p12 amplification, 19q12 amplification, 16p13.3 deletion and 17p13.1 deletion; In 30 pediatric patients, 292 CNVs we detected including 277 amplifications and 15 gene homozygous deletions, and the most frequent CNVs included 1p36.13 amplification, 6p21.1 amplification, 12q14.1 amplification, and 17p11.2 amplification; In 14 adult patients, 118 CNVs were detected including 102 amplifications and 16 gene homozygous deletions, and the most common CNVs included 8p12 deletion, 9p21.3 deletion, 13q13.3 deletion, 15q15.1 deletion, and 16p13.3 deletion (**Figure 3B**).

### 3.5 Signature analysis

**Figure 4:**
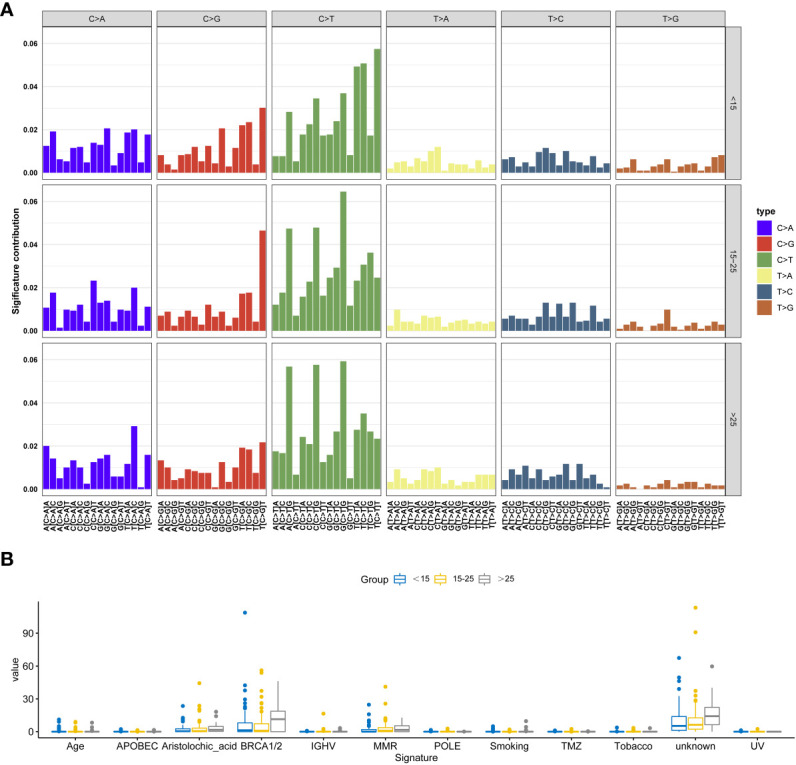
Signature analysis of this cohort. **(A)** The most significant signatures in adolescent osteosarcoma patients. **(B)** The most significant signatures in adult osteosarcoma patients.

We observed similar trends across all six possible point mutation types in the three groups. C>T mutations were the most abundant types, followed by C>A and C>G. Upon conducting a comprehensive Signature analysis, underscores the importance of age as a factor in mutational processes. The differential signature analysis indicates that mutations characteristic of BRCA1/2 and Aristolochic acid are more pronounced in adults. The individual distribution of these signatures was shown in **Figure 4** and **Table S1**.

### 3.6 Prognosis analysis of osteosarcoma patients

**Figure 5:**
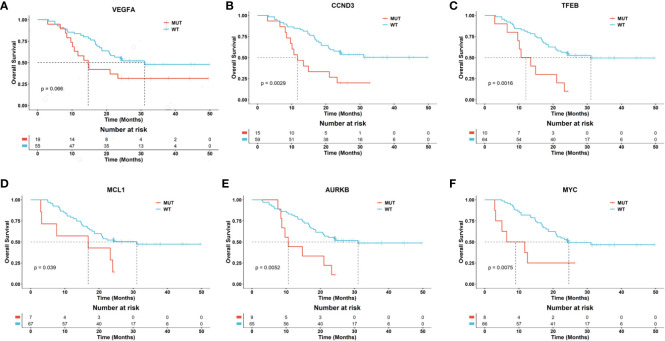
Potential biomarkers related to the prognosis of osteosarcoma patients. The Kaplan-Meier curves separately depict the overall survival (OS) for patients with and without mutations in the genes VEGFA **(A)**, CCND3 **(B)**, TFEB **(C)**, MCL1 **(D)**, AURKB **(E)**, and MYC **(F)**. The p-values between the risk groups were calculated using the log-rank test.

Based on the genomic alteration, we evaluated the prognostic significance of specific genetic alterations in a cohort of patients. We collected the effective overall survival (OS) information of 74 patients, including 39 pediatric, 31 adolescents and 4 adult patients. The overall median DFS was 24 months. Notably, mutations in the genes *MCL1*, *MYC*, *TFEB*, *CCND3*, *AURKB*, and *ALOX12B* were associated with a significantly worse prognosis, with p-values less than 0.05, underscoring their potential role as markers for aggressive disease courses. On the other hand, mutations in VEGFA were also correlated with a poorer prognosis, with p-values approaching the threshold of significance (near 0.05), suggesting a trend that warrants further investigation for definitive conclusions (**Figure 5** and **Table S2**).

### 3.7 Available drug mutations in osteosarcoma

**Figure 6:**
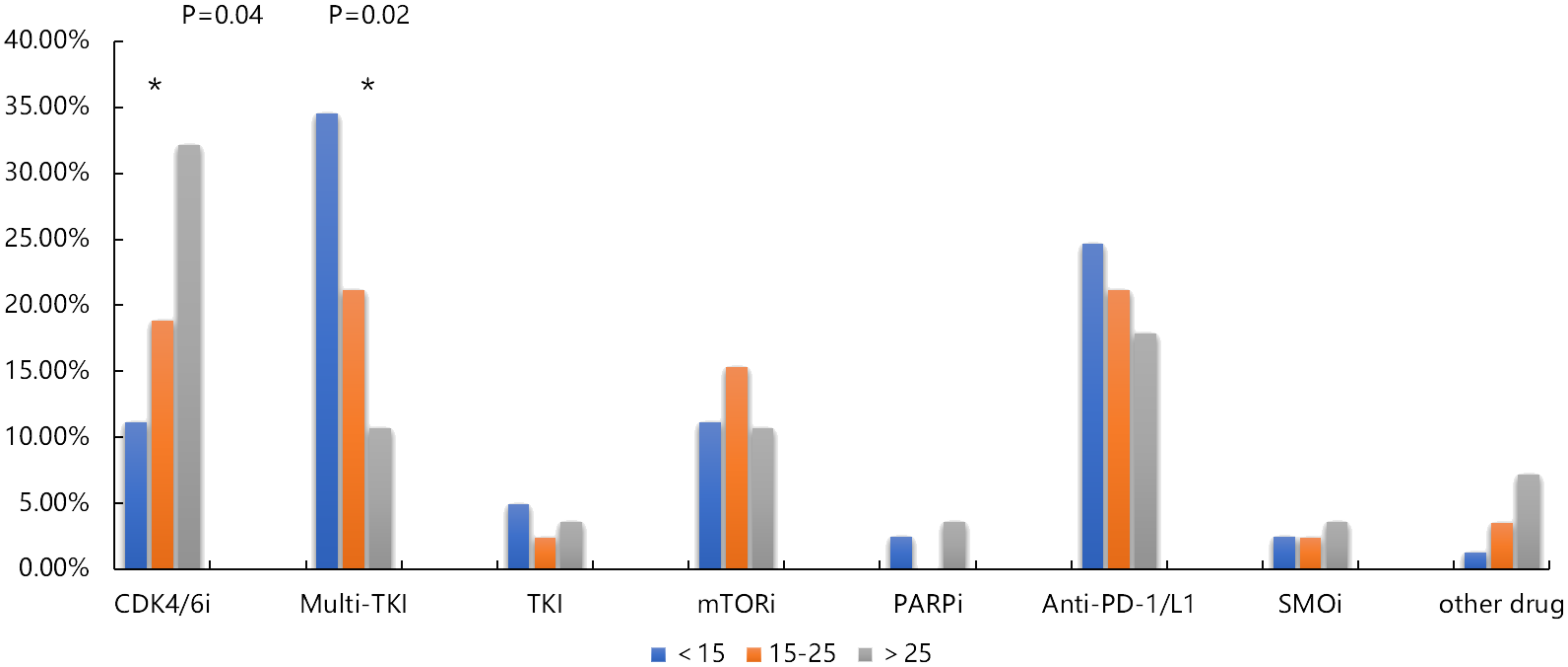
Comparison of available drug mutation distribution in pediatric, adolescent and adult osteosarcoma group. * p < 0.05.

Actionable drug mutations were detected in 58% (113 out of 194) of patients, encompassing 185 mutations across 35 target genes, with *VEGFA* being the most frequently mutated gene associated with drug responsiveness. About 25% (49/194) of patients harbored the mutations of angiogenesis related gene amplification, such as *VEGFA*, *KIT*, *KDR*, *PDGFRA*, *ARAF*, *FGFR1*, *VHL*, and *TFE3*, and 18% (34/194) patients were harbored CDK4/6 inhibition related mutations. These results suggested the potential targeted treatment of osteosarcomas. In pediatric adolescent patients, 86 available drug mutations from 23 targeted genes were detected in 49 osteosarcoma patients. In adolescent patients, 66 available drug mutations from 25 targeted genes were detected in 49 osteosarcoma patients, and in adult patients, 25 available drug mutations from 15 targeted genes were detected in 15 osteosarcoma patients. The most common available drug mutated genes include *VEGFA* in pediatric and adolescent patients, *CDK4* in adolescent patients, and *CDKN2A*, and *CDKN2B* in adult patients. According to the drug type, the drug available mutations were divided into Anti-PD-1/PD-L1 related mutations such as *CD274* and *PDCD1LG2*, *CDK4*/*6* inhibitor related mutations such as *CDKN2A*, *CDKN2B*, *CCND1*, and *CDK4*, mTOR inhibitor related mutations such as *PTEN*, *MTOR*, *FBXW7*, *PIK3CA*, and *STK11*, Multi-TKI related mutations such as *VEGFA*, *KIT*, *KDR*, *PDGFRA*, *ARAF*, *FGFR1*, *VHL*, and *TFE3*, *VEGFA*, *KIT*, *KDR*, *PDGFRA*, *ARAF*, *FGFR1*, *VHL*, and TFE3, and PARP inhibitor related mutations such as *ATM*, *BRCA2*, and *BRCA1*, and TKI related mutations such as *ALK*, *KRAS*, *NRAS*, *ROS1*, *MET*, *BRAF*, *EGFR*, and *MET*. We found that the frequency of Multi-TKI related mutations was significantly higher in pediatric and adolescent patients than that in adult patients (34.57% vs 21.18% vs 10.71%, P =0.02), while the frequency of CDK4/6 inhibitor related mutations was significantly higher in adult patients than that of pediatric and adolescent patients (32.14% vs 18.82 vs 11.11%, P= 0.04) (**Figure 6**).

In the cohort of patients with *VEGFR* amplification, 13 individuals underwent adjuvant therapy with multi-kinase inhibitors post-surgery, including agents such as anlotinib, apatinib, and recombinant human vascular endothelial inhibitors. The median duration of follow-up was 3 months (1-20 months). Within this subgroup, 3 patients achieved a Partial Response (PR), all of whom were younger than 15 years. Furthermore, 4 patients maintained Stable Disease (SD), and 6 patients experienced Progressive Disease (PD). Notably, among the patients with PD, 4 exhibited additional genetic alterations: 2 cases with *CDK4* amplification, 1 with *PIK3CA* amplification, and 1 with a *PTEN* mutation.

One notable case of PR was a 13-year-old patient diagnosed with proximal tibial osteosarcoma, which demonstrated *VEGFR* amplification. The patient underwent a successful surgical resection of the tumor on December 18, 2018, which was classified as Stage IIB by postoperative pathology. Between January 14, 2019, and February 22, 2019, the patient received two cycles of adjuvant chemotherapy using pirarubicin and cisplatin. Subsequent treatment from April 2019 to January 2020 included the oral *VEGFR* inhibitor anlotinib hydrochloride (Focilex).

**Figure 7:**
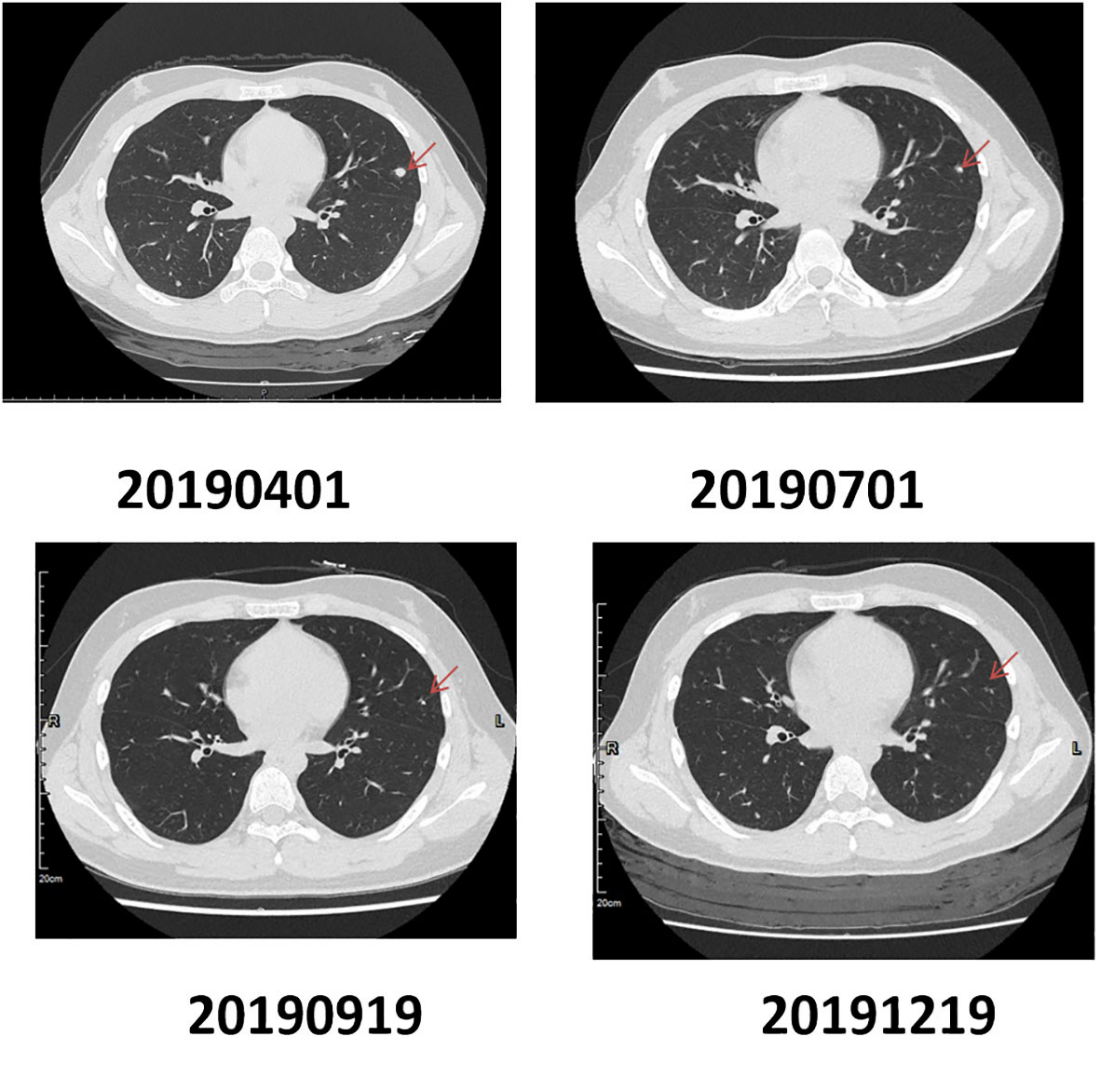
Computed Tomography (CT) imaging changes in tumor size during patient medication regimen.

To assess treatment response, a baseline imaging study was obtained before the initiation of anlotinib, which identified multiple lung nodules. According to RECIST criteria, target lesions were defined, with the largest nodule in the lingular segment of the left upper lobe measuring 10mm in diameter (considered as the target lesion). Follow-up CT scans were performed to evaluate the response at regular intervals. On July 1, 2019, the size of the target lesion had decreased to 5mm, and by September 20, 2019, it had further reduced to 4mm. Comparative imaging on December 19, 2019, showed continued reduction in the size of the target lesion as well as non-target lesions. As of March 18, 2020, the target lesions have further reduced in size. Based on the RECIST criteria, this patient achieved a Partial Response (PR), with a significant reduction in the size of the target lesion and overall tumor burden (**Figure 7**).

## 4 Discussion

With the development of molecular biology and next-generation sequencing technology, the genomic variation information of many cancer types has been revealed and applied to targeted therapy. In osteosarcoma, due to the lack of osteosarcoma samples and extensive genomic heterogeneity, the determination of its somatic therapeutic targets is particularly challenging. Although germline genetic variation is an important risk factor associated with osteosarcoma, many studies are still aiming to explore new treatments by identifying osteosarcoma related biologically important pathways (1, 25, 26). In this study, we collected 194 osteosarcoma patients, grouped them according to their age, and analyzed the mutation characteristics of pediatric, adolescent and adult osteosarcoma patients.

Previous studies have shown that the common mutant genes in osteosarcoma include *TP53*, *RB1*, *MYC* (6, 7), however, there are few studies on the mutation characteristics of osteosarcoma patients based on large samples. Our results showed that not only identified the high-frequency mutations of *TP53* and *RB1*, but also revealed high-frequency mutations in *FLCN*, *NCOR1*, *VEGFA*, *CCND3*, *TFEB*, *MAP2K4*, and *ATRX* in osteosarcoma. Mutations in *FLCN* gene are associated with Birt-Hogg-Dube syndrome, which is characterized by fibrofolliculomas, renal tumors, lung cysts, and pneumothorax (https://www.ncbi.nlm.nih.gov/gene/201163). The *FLCN* gene is commonly observed in patients diagnosed with both primary and metastatic osteosarcoma (27), with a higher frequency of alterations seen in pediatric cases compared to those in adults (28). *NCOR1* is a tumor suppressor gene. According to AACR Genie cases, *NCOR1* amplification is frequently occurred proportion in osteosarcoma, which only less frequent than leiomyosarcoma (29) (https://www.mycancergenome.org/content/gene/ncor1/). *NCOR1* has been reported to be associated with prognosis in many cancer type (30, 31). In bladder cancer, *NCOR1* mutation is associated with immune biomarker such as TMB, suggesting that it may be used as biomarker for immunotherapy (32). Therefore, *NCOR1* has great potential for clinical application. Its high frequency amplification mutation in osteosarcoma may become a potential biomarker for guiding the treatment. *ATRX* deficiency can promote tumorigenesis, including enhanced cell movement of glioma cells and *TGF* in hepatoma cells- β Activation and *CDH1* (E-cadherin) down-regulation (33–35). In osteosarcoma, *ATRX* deficiency also can promote tumor formation, growth, infection and metastasis (36). In this study, there was no significant difference in the proportion of *ATRX* mutations between pediatric, adolescents and adults.

The onset characteristics of osteosarcoma are related to the age of patients. The first peak of osteosarcoma is in adolescence, which indicates that there is a close relationship between adolescent growth spurt and osteosarcoma; The second peak of osteosarcoma occurs in adults over 65 years old, which may be related to the accumulation of mutations and the occurrence of diseases (37). However, little is known about the mutation characteristics of pediatric, adolescent and adult patients with osteosarcoma. The comparison of mutation characteristics between the three groups suggested that pediatric, adolescents are more prone to gene amplification / deletion and fusion than adults, and adults may accumulate more point mutations over time. The differential signature analysis indicates that mutations characteristic of *BRCA1/2* and Aristolochic acid are more pronounced in adults, and homologous recombination deficiency associated signature may lead to the accumulation of DNA damage and genomic instability (38). These results suggested the special molecular characteristics of pediatric, adolescent and adult osteosarcoma patients. Compared to adult patients with osteosarcoma, we observed that the amplification frequencies of *NCOR1*, *VEGFA*, *CCND3*, and *TEEB* were higher in pediatric and adolescent patients. In contrast, the amplification frequencies of *CDK4*, *TSPAN31*, *GLI1*, *MDM2*, and *FRS2* were highest in adult patients with osteosarcoma, followed by adolescents, and lowest in pediatric patients. There have been few reports on mutations of *TEEB*, *GLI1*, *MDM2*, and *FRS2* in osteosarcoma. This suggests that the genetic mutation characteristics of osteosarcoma are age-related, and the pathogenesis of osteosarcoma may differ across various age groups. The *CCND3* encodes a highly conserved cell cycle regulatory protein, and the expression of cyclin is characterized by cyclicity. The fusion of *KCNMB4-CCND3* was ever detected in osteosarcoma and promoted the migration of osteosarcoma cell line SAOS-2 (39). The overexpression of *VEGFA* in various human tumors is associated with tumor cell invasion, increased vascular density, tumor metastasis, tumor recurrence and poor prognosis (40). The amplification of *VEGFA* may elevate their respective proteins in osteosarcoma (41). In this study, we grouped osteosarcoma patients by their age and suggested that high frequency *VEGFA* and *CCND3* amplification may be the molecular characteristics of adolescent osteosarcoma patients. There has been an increasing interest in exploring the role of *TSPAN31* in cancerous diseases. Earlier studies have discovered a significant rise in *TSPAN31* levels in osteosarcoma, hinting at its possible link to the expansion and dissemination of the tumor. It is also important to point out that *TSPAN31* acts as a natural antisense transcript to *CDK4*. Investigations have demonstrated that *TSPAN31* plays a role in the advancement of tumors by controlling the expression of *CDK4*, with evidence from research on cervical and liver cancer cases (42–44). *FRS2* and *MDM2* are close to each other on chromosomes and are often detected in the form of co-amplification. Previous study had shown that the amplification of *FRS2* and *MDM2* occurs frequently in low-grade osteosarcoma (95%, 21/22), and only one of these patients is younger than 20 years old (45). Our research underscores the high amplification frequency of MDM2 within the adult patient population, particularly considering MDM2's role in the negative regulation of the p53 pathway—a pivotal aspect of tumor biology. The amplification of MDM2 correlates with increased protein levels capable of inhibiting p53, thereby promoting tumor progression and potentially exerting a detrimental effect on patient outcomes. The age-specific prevalence of MDM2 amplification identified in our study positions it as a valuable biomarker for the diagnosis and prognosis of adult osteosarcoma. It may also signal the need for more aggressive therapeutic approaches in this demographic group. Furthermore, the potential for targeted therapy against MDM2, with inhibitors that are already undergoing clinical trials for other cancers, presents an optimistic path for personalized treatment strategies. Recognizing the necessity for additional validation, we propose further research to solidify the predictive and prognostic utility of MDM2 amplification. This would involve larger patient cohorts and an analysis of clinical outcomes, such as treatment response, disease progression, and survival rates. Other studies have shown that higher TMB is more distributed in older patients (46). Similar results have been obtained in our study. The TMB of adult patients is higher than that of pediatric patients, which may be related to the long-term accumulation of point mutations. This also supports our previous conjecture to properly explain the age characteristics of osteosarcoma patients. A significant role of NGS in cancer precision medicine is to detect potential available targets (47). *PDGFRA* and *KIT* mutations are common in gastrointestinal stromal tumors (48). Our results suggest that these two mutations are also common in patients with osteosarcoma. Sunitinib, regorafenib, and imatinib have kinase inhibitory activities of *KIT* and *PDGFRA* (49). Studies have shown that regorafenib is also active in osteosarcoma (50). However, we did not find any difference in the frequency of these gene mutations between adolescent and adult osteosarcoma patients.

Osteosarcomas are genetically defined by significant genomic instability, which is manifested through extensive aneuploidy, the existence of imbalanced chromosomal rearrangements, and the frequent amplification or deletion of various genomic segments. Previous studies have indicated that amplifications in osteosarcoma are commonly concentrated in specific regions such as regions 6p12-p21, 8q24, and 17p11.2-p12 (51–53). However, differences in amplification regions among patients of varying age groups have not yet been reported. Our genomic analysis identified a chromosomal hotspot harboring high-frequency mutations at the 6p21 locus, particularly involving the *TFEB*, *CCND3*, and *VEGFA* genes. This region is known to be critical during pediatric and adolescence, and the presence of mutations in this area may indicate a predisposition to certain malignancies in younger populations. These results indicate the unique prognosis molecular indicators of osteosarcoma patients in different age groups.

Previous studies have indicated that the loss of *RB1*, amplification of *MYC*, and amplification of *VEGFA* are molecular characteristics that can identify high-risk patients. However, these risk factors have not been fully validated and cannot yet serve as the basis for clinical risk stratification (54). Our research findings suggest that in addition to *MYC*, mutations in the genes *MCL1*, *TFEB*, *CCND3*, *AURKB*, and *ALOX12B* are also significantly associated with poor prognosis. Additionally, mutations in the *VEGFA* gene have shown a trend towards association with poor prognosis, although the p-value is close to the significance threshold and has not yet reached statistical significance. Given the critical role of *VEGFA* in angiogenesis, this trend suggests that the gene might play a role in vascular supply and tumor microenvironment formation in osteosarcoma. These findings prompt us to further investigate the specific role of *VEGFA* in the development of osteosarcoma and to explore its potential as a therapeutic target.

*VEGFA* can bind to vascular endothelial growth factor receptor on the surface of cell membrane, produce biological effects through a series of signal pathways, and finally lead to angiogenesis (55), and plays a role in regulating vascular development and is the target of targeted drugs to inhibit angiogenesis (56). VEGF/VEGFR can be used as a target to inhibit angiogenesis (57–59). The most common CNV of osteosarcoma includes *VEGFA*, which suggested the potential opportunity to benefit from anti-angiogenic agent (60). In our cohort, we stratified the patients and found that 100% of patients with *VEGFA* mutations belong to pediatric and adolescent patients. The *VEGFA* variations correspond to Multi-TKI, and the high proportion of *VEGFA* variants in pediatric and adolescents may result in the more opportunities to benefit from Multi-TKI treatment than adults. This also showed that it is necessary to stratify patients with osteosarcoma according to their age and provide accurate medical treatment.

The CDK4/6 inhibitor related gene include *CDKN2A/B*, *CCND1*, and *CDK4*, and the variations frequency of these genes increase with age (61). Research has indicated that elevated levels of *CDK4* expression and its amplification in tumors serve as predictive biomarkers for resistance to standard chemotherapy in osteosarcoma (OS) patients, suggesting that palbociclib holds potential as an effective treatment option for this clinically challenging group (62). Our study findings confirm the same trend, with higher frequencies of *CDKN2A/B*, *CCND1*, and *CDK4* gene mutations observed in adults. This indicates that with the growth of age, adult osteosarcoma patients are more likely to accumulate age-related gene mutations, including *CDKN2A/B*, *CCND1*, and *CDK4*, which supports that adult osteosarcoma patients may have more opportunities to benefit from CDK4/6 inhibitors.

Additionally, our study found that the number of patients under the age of 15 (81 cases) closely matches those aged 15-25 (85 cases), with their molecular differences predominantly centralized in variations in enriched signaling pathways. In patients older than 15, gene mutations are mainly enriched in the cell cycle and PI3K/mTOR signaling pathways. In contrast, patients younger than 15 exhibit gene mutations largely concentrated in the cell cycle and angiogenesis signaling pathways. CDK4 mutations are more frequently observed in adolescent patients. Furthermore, all patients who achieved partial remission with multitargeted kinase inhibitor therapy were under the age of 15. There are also mutual molecular features; mutations in VEGFA, CCND3, and TFEB are significantly present in both children and adolescents. The amplification rates of VEGFA and XPO5 are high in pediatric and adolescent patients as well, whereas mutations in CDKN2A and CDKN2B are more common in adults, underscoring the stark differences between pediatric/adolescent and adult patients. Adolescent patients may represent a transitional state in terms of molecular characteristics and treatment responses, falling between children and adults.

The study examining molecular distinctions among pediatric, adolescent, and adult patients with osteosarcoma is subject to several limitations: It encompasses a modest patient population of 194 cases, potentially curtailing the power to discern differences and possibly not reflecting the wider osteosarcoma demographic. Although the research indicates potential associations between certain mutations and poorer outcomes, these findings do not confirm causality or establish definitive prognostic indicators without additional substantiation. Consequently, while the study sheds valuable light on the molecular landscape of osteosarcoma across various age groups, the results warrant cautious interpretation and require corroboration via more extensive, prospective, multicentered studies with prolonged follow-up periods and randomized clinical trials to determine the true clinical relevance of the molecular markers identified. Another limitation of our study is that 28 of 194 patients who were enrolled had tissue samples obtained from needle biopsies, and tumor heterogeneity poses substantial challenges. This heterogeneity implies that distinct molecular profiles may be present in different tumor areas, making genetic data interpretation complex. Biopsy samples inherently offer a limited snapshot of the entire tumor since they are typically obtained from a single region. This selective sampling might render an incomplete or potentially misleading picture of the tumor's genetic makeup. The impact of tumor heterogeneity on study outcomes is significant; it may lead to overrepresentation of genetic alterations that are prevalent in the sampled region but not indicative of the whole tumor. Conversely, crucial mutations in other tumor parts may be overlooked. Such omissions could profoundly affect our conclusions on the correlation between genetic mutations and clinical outcomes, as they might not accurately reflect the mutational burden of the entire tumor. To mitigate the potential biases introduced by tumor heterogeneity in biopsy samples, future studies should consider incorporating multiple biopsy samples from diverse tumor regions when possible. Additionally, the comparison of genetic data from biopsy samples with that from complete tumor resections could yield a more holistic view of the molecular changes in osteosarcoma. Employing standardized methods for sample collection and DNA sequencing, as well as including larger patient cohorts from various demographics, is essential for future research. These steps will help lessen the effects of heterogeneity and enhance the generalizability of the study's findings to a broader osteosarcoma patient population. In this study, the majority of the specimens were surgical samples obtained after 2 to 3 cycles of neoadjuvant chemotherapy. We acknowledge that neoadjuvant chemotherapy may impact the genomic analysis of the surgical tissue. The potential effects include a significant reduction in the number of detectable tumor cells, which could affect genetic analyses that require a sufficient quantity of tumor cells. Chemotherapy might selectively eliminate tumor cell populations that are sensitive to the drugs, leaving behind resistant populations, which could result in altered genetic characteristics of the tumor. Furthermore, chemotherapy could influence the gene expression patterns within tumor cells, leading to the upregulation or downregulation of certain genes, thereby affecting the outcomes of genetic testing.

In this study, we examined the molecular characteristics of osteosarcoma across pediatric, adolescent, and adult patients, highlighting differences in the molecular mechanisms of the disease between adolescents and adults, and investigated biomarkers that could specifically predict the prognosis of adolescent osteosarcoma patients. Furthermore, we identified the importance of age in the distribution of copy number variations (CNVs) and point mutations, revealing age-related pathogenic differences and the potential influence of certain genetic variations on patient outcomes. Notably, younger patients with VEGFR-amplified osteosarcoma who received adjuvant multi-kinase inhibitor therapy achieved partial remission or disease stabilization, emphasizing the necessity for personalized treatment approaches. These insights contribute to a deeper understanding of the genetic heterogeneity in osteosarcoma and support the development of precise diagnostic and treatment strategies that are tailored to the individual needs of patients from different age cohorts.

## Data availability statement

The authors acknowledge that the data presented in this study must be deposited and made publicly available in an acceptable repository, prior to publication. Frontiers cannot accept an article that does not adhere to our open data policies.

## Ethics statement

The studies involving humans were approved by The Clinical Research and Laboratory Animal Ethics Committee of the First Affiliated Hospital of Sun Yat-sen University. The studies were conducted in accordance with the local legislation and institutional requirements. The human samples used in this study were acquired from Tissue samples are the remaining samples for pathological diagnosis, which will not affect the subject's diagnosis. The studies were conducted in accordance with the local legislation and institutional requirements. The human samples used in this study were obtained in compliance with ethical standards. Written informed consent for participation was not required from the participants or the participants' legal guardians/next of kin in accordance with the national legislation and institutional requirements. Written informed consent was obtained from the minor(s)' legal guardian/next of kin for the publication of any potentially identifiable images or data included in this article.

## Author contributions

JS: Funding acquisition, Project administration, Resources, Supervision, Writing – review & editing. CZ: Data curation, Funding acquisition, Writing – review & editing. RH: Conceptualization, Data curation, Investigation, Writing – original draft. TL: Investigation, Supervision, Writing – review & editing. YW: Formal analysis, Writing – original draft. JT: Resources, Supervision, Writing – review & editing. LZ: Formal analysis, Visualization, Writing – review & editing. BW: Investigation, Methodology, Writing – review & editing. JH: Formal analysis, Methodology, Writing – review & editing. ZZ: Resources, Validation, Writing – review & editing. XX: Resources, Supervision, Writing – review & editing. GH: Data curation, Resources, Writing – review & editing. KW: Resources, Writing – review & editing. JY: Project administration, Resources, Supervision, Validation, Writing – review & editing.

## Supplementary material

Supplementary Figure 1 Distribution of mutation types in osteosarcoma patients.
