# Supplementary figures and images for: Age-dependent molecular variations in osteosarcoma: implications for precision oncology across pediatric, adolescent, and adult patients
Source: Front Oncol. 2024 May 22;14:1382276. doi: 10.3389/fonc.2024.1382276 (PMC11150704; doi:10.3389/fonc.2024.1382276)

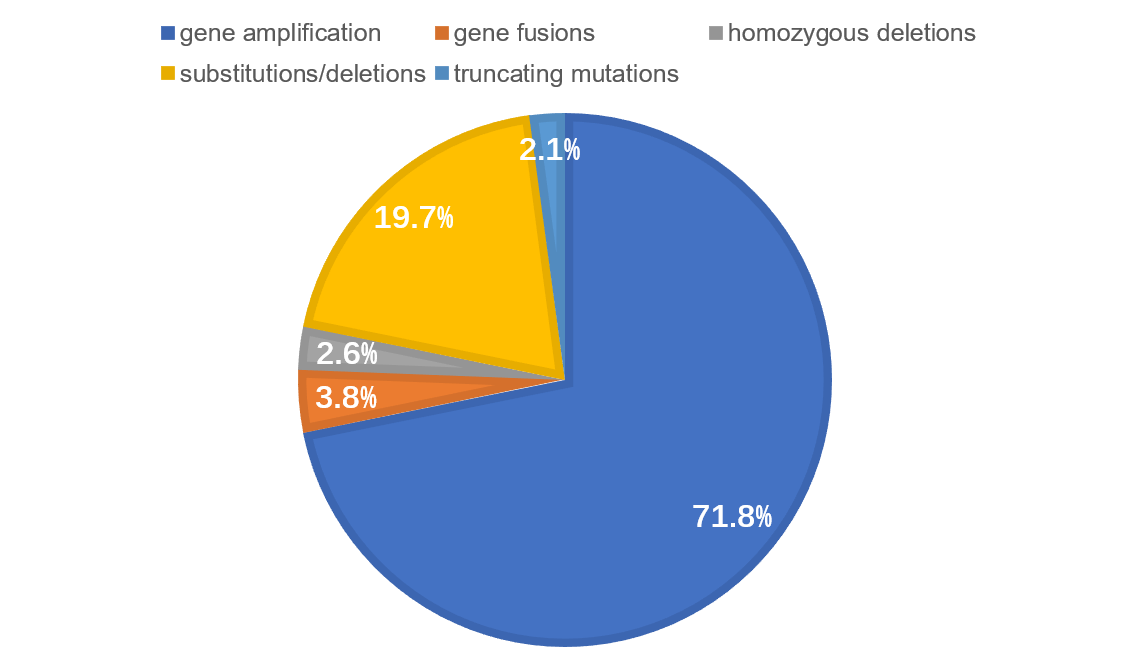

Supplement: Supplementary Figure 1 — Distribution of mutation types in osteosarcoma patients. [file Image_1.tif]
